# Model-Driven Decision Making in Multiple Sclerosis Research: Existing Works and Latest Trends

**DOI:** 10.1016/j.patter.2020.100121

**Published:** 2020-11-13

**Authors:** Rayan Alshamrani, Ashrf Althbiti, Yara Alshamrani, Fatimah Alkomah, Xiaogang Ma

**Affiliations:** 1 Department of Computer Science, University of Idaho, Moscow, ID 83844-1010, USA; 2 Department of Information Technology, Taif University, Taif, Makkah 26571, Saudi Arabia; 3 INTO Program, Washington State University, Pullman, WA 99164-3251, USA; 4 Department of Information Systems, Princess Nourah Bint Abdulrahman University, Riyadh 11671, Saudi Arabia

**Keywords:** multiple sclerosis, shared decision making, decision support systems, semantic web, ontology, knowledge-based systems, machine learning

## Abstract

Multiple sclerosis (MS) is a neurological disorder that strikes the central nervous system. Due to the complexity of this disease, healthcare sectors are increasingly in need of shared clinical decision-making tools to provide practitioners with insightful knowledge and information about MS. These tools ought to be comprehensible by both technical and non-technical healthcare audiences. To aid this cause, this literature review analyzes the state-of-the-art decision support systems (DSSs) in MS research with a special focus on model-driven decision-making processes. The review clusters common methodologies used to support the decision-making process in classifying, diagnosing, predicting, and treating MS. This work observes that the majority of the investigated DSSs rely on knowledge-based and machine learning (ML) approaches, so the utilization of ontology and ML in the MS domain is observed to extend the scope of this review. Finally, this review summarizes the state-of-the-art DSSs, discusses the methods that have commonalities, and addresses the future work of applying DSS technologies in the MS field.

## The Bigger Picture

Multiple sclerosis (MS) is a disorder that strikes the central nervous system of the human body. This article reviews state-of-the-art decision support systems (DSSs) in MS research, as recent studies have highlighted the importance of DSSs in the medical realm. However, the utilization of decision support systems for MS remains an open challenge. A special focus in this article is given to model-driven DSSs, which uses knowledge representation to simplify the complex process for decision makers. We find that most investigated studies use knowledge-based and machine learning approaches. Based on the literature review, we suggest some future work of applying DSSs in the MS domain. Potential future directions should focus on applying DSS technologies to understand the MS patterns, etiology, effects on the quality-of-life, and correlations with other disorders.

## Teaser

Multiple sclerosis (MS) is a disorder that strikes the central nervous system of the human body. This article reviews state-of-the-art decision support systems (DSSs) in MS research. A special focus is given to model-driven DSSs, which uses knowledge representation to simplify the complex process for decision makers. We find that most investigated studies use knowledge-based and machine learning approaches. Based on the literature review, we suggest some future work of applying DSSs in the MS domain.

## Introduction

Multiple sclerosis (MS) is a chronic neurological disorder that stimulates the immune system to attack the central nervous system of the human body.^1^, ^2^, ^3^ Genetic and environmental causes are the possible triggers of MS while exposure to the Epstein-Barr virus, vitamin D deficiency, and smoking habits are likely factors that enable the progression of MS.^4^ However, the exact causes of MS are still unknown. MS patients may experience several symptoms independently or concurrently during the course of the disease, such as sensory, visual, motor, cognitive, and cerebellar disorders.^5^ MS affects several millions of people around the globe, especially young adults.^6^ In general, men are less likely to develop MS compared with women.^7^ Four medical terms represent MS in terms of the progression level: relapsing-remitting MS (RRMS), secondary-progressive MS (SPMS), primary-progressive MS (PPMS), and progressive-relapsing MS.^4^ The MS diagnostic procedure requires neurological examinations, such as magnetic resonance imaging (MRI), lumbar punctures (LP), and blood tests to confirm MS cases.^8^ In addition, neurologists prescribe existing therapies to control the symptoms and the progression of MS as it turns out that this disorder cannot be cured or prevented.^9^ Thus, neither the treatment nor the diagnosis of MS is easy. This is because MS shares several clinical features with other diseases and has no consensus approach in MS diagnosis.^6^ In fact, the decision making in both treatment and diagnosis of MS is critical and relies heavily on the experience and the judgment of the neurologist. Therefore, the quality of decisions for that matter remains doubtful due to the presence of uncertainties associated with MS. Moreover, MS is a preference-sensitive condition, so both the physician and the patient participate in the decision-making process, i.e., shared decision making.^10^ Hence, this would impose a great responsibility upon the contributors in the decision-making process as they must have full knowledge about the current state of the condition and the potential risks and benefits of all possible options to achieve the optimal decision. Comprehensively, it would be beneficial to have easy-to-use automated solutions that could propose several optimal alternatives to make the shared decision making easier for all participants in this process.

Decision support systems (DSSs) are computer-based systems devoted to people who are concerned with decision making so they could solve real-world problems via worthy decisions.^11^ DSSs are largely accepted in modern commercial businesses and have been accomplishing significant successes.^12^ DSSs in the medical realm are very promising, especially for enhancing the decision-making process. Recent studies demonstrate the increasing importance of DSSs in medicine, i.e., clinical decision support systems (CDSSs), for helping intended decision makers to nominate the right decision among several alternatives as often as possible.^13^ DSS technologies are potentially favorable tools in the MS domain. For instance, DSSs could provide decision makers with useful information (e.g., alerts, warnings, or predictions) about MS cases.^14^ The benefits of using DSSs in the MS domain include: enabling access to neurologists, enhancing clinical documentation and prescription processes, escalating diagnosis accuracy, minimizing time loss and healthcare expense, enhancing diagnostic predictions, maximizing the quality of patients’ lives and care provided, and improving the quality of decisions.^1^^,^^15^ However, with all these benefits, the utilization of DSSs in the MS field is not encouraging. To the best of our knowledge, aside from this review, the investigation of the DSSs in the MS domain is still insufficient because there is no published review on the presented topic.

The goal of this paper is to analyze the state-of-the-art DSSs in MS research. This work answers the following questions: (1) What DSSs are currently used in the MS domain? (2) What are their key fundamental methodologies? (3) What are they used for? (4) What are the current most promising technologies associated with decision making in the MS domain? Answering these questions would (1) demonstrate the importance of adopting DSSs in MS research and (2) show the extent of technologies, mostly correlated with DSSs, adopted for decision-making purposes in the scope of MS (definition of concepts and details of methods are explained below in subsections “The Uses of Ontology in MS Research” and "The Utilization of Machine Learning in MS Studies").

A model-driven DSS is a type of DSS that uses complex and quantitative models that provide a simplified and straightforward knowledge representation to decision makers.^16^ Model-driven DSSs are distinguished by two characteristics: (1) a model in a model-driven DSS is made accessible to experts with no technical background, and (2) DSSs of this type are reusable in equivalent decision situations.^16^ Definitely, both the data science community and the MS community need to have a clear overview of the current state of the automated tools used to support decision making in the MS domain. This review is directed to data scientists, especially those who are interested in complex modeling approaches within the health informatics field. This review demonstrates the current DSS technologies within the domain of MS research, so data scientists can glance over the recent trends and the potential future research paths to enrich this research field with new automated decision-making technologies. Surely, MS community members and MS specialists will find useful information about the most recent technologies that could help them in their daily clinical practices.

The remainder of this paper is organized as follows: the next section explains the methodology conducted to acquire the needed resources. The following two sections (1) describe the results in accordance with the analysis methodology and (2) discuss remarkable findings and trends. The final section concludes this study.

## Literature Search Methodology

All relevant articles used to carry out this literature review were collected from six database sources: Google Scholar, DBLP Computer Science Bibliography, Web of Science, PubMed, ACM Digital Library, and IEEE Xplore Digital Library. The searching strategy utilized Multiple Sclerosis, Decision Support, Ontology, Semantic Web, Machine Learning, and Knowledge Graph as the search phrases, where the first phrase (i.e., MS) is combined with each of the latter ones (i.e., technical terms) with the logical operator “AND” to form a searching keyword. Each keyword was used to retrieve articles that observe current trends of MS research with the help of the technical term noted in the keyword. This is done to determine if there are correlations in MS research between the retrieved articles and the use of DSSs or the decision-making process in the MS domain. Articles published in English between 2007 and 2019 were collected during September and December 2019 and were screened out. Narrowing the number of the selected articles took two steps. First, the abstract (along with the title) of each article was analyzed to compose a subset of articles corresponding to the searching keywords. Subsequently, the full text of each article in the composed subset was resolved to report the most interesting articles that cope with the inclusion and exclusion criteria applied to the searching strategy of this literature review.

**Figure 1:**
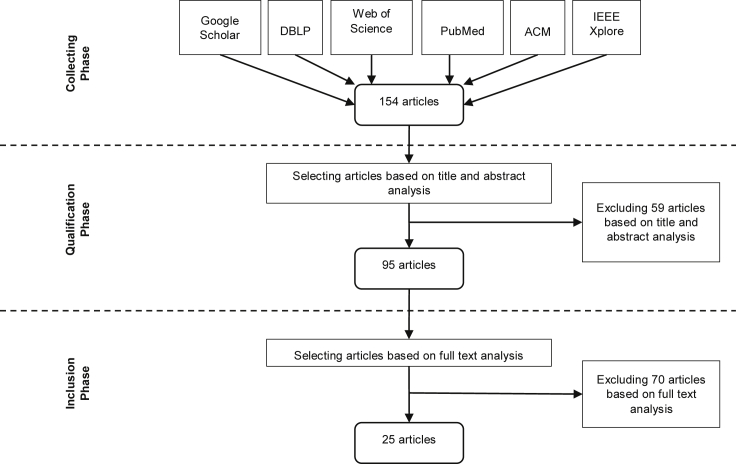
Sources and Steps in the Literature Selection Process

Articles reviewed in this work comply with two inclusion criteria. The first criterion ensures that the machine-driven model applied in an article has a detailed description of its functionality. The second criterion justifies that the whole purpose of an article being analyzed is for diagnosing, treating, classifying, or predicting MS specifically. Research papers that have pure medical knowledge about MS and others that use solid mathematical theories with no automated models or running computer systems are therefore excluded. Likewise, papers that are dedicated to general neurology, except the ones that have MS as an example or as a case study, are excluded as well. The acquired data from each article consists of the author, the main intention of the paper, dataset information, system in use (if applicable), applied model and algorithms (i.e., research methodology), outcomes and remarks, and evaluation approaches and results (if reported). Figure 1 briefly illustrates the literature selection process.

## Literature Analysis and Results

Using the searching strategy presented earlier, a total of 154 articles were retrieved from several electronic databases (see Figure 1). After scanning through the article abstracts, the list was narrowed down from 154 to 95 articles. After the full-text analysis phase, 25 of the 95 articles were selected based on the inclusion and the exclusion criteria. The overall objective of each of the selected research papers was determined for categorizing them into subgroups. It should be noted that one article could belong to more than one category, but it was categorized under the subgroup that was the most appropriate given the goal of that article. The following subsections outline the main relevant methods and provide examples of how technologies related to DSSs and decision-making processes are implemented for the MS domain.

### The Role of DSSs in MS Quest

Healthcare organizations are increasingly in need of DSSs, namely CDSSs, that are understandable by non-technical audiences, such as healthcare providers. CDSSs aid clinical decision making by providing practitioners with insight knowledge and information about their patients for generating suitable assessments or recommendations.^17^ In the MS domain, the use of DSSs, or CDSSs, is just as important as their use in other healthcare areas. Indeed, the existing DSS technologies tied with MS, as explained next and summarized in Table 1, are for the sake of classifying, diagnosing, predicting, or treating MS.

**Table 1:** Summary of the Analyzed MS DSSs Articles

Author(s)	Year	Objective				Methodology Basis	System in Use
		Classification	Diagnosis	Prediction	Treatment		
Esposito and De Pietro^18^	2011	✓				Knowledge-based	
De Falco et al.^19^	2016	✓				DE	
Siddiqui et al.^20^	2015	✓				DWT, PCA, and LS-SVM	
Esposito et al.^21^	2011	✓				Knowledge-based	
Linder et al.^22^	2009		✓			MLR and ANN	CAD tool
Pourakbari et al.^23^	2014		✓			Image processing	
Dogan and Duru^24^	2011		✓			SVM and k-means	
Almasi et al.^25^	2015		✓			Case-based reasoning and rule-based reasoning	
Veloso^26^	2013			✓		Agent-based simulation model	
SLCMSR^27^	2007			✓		OLAP tool uses matching algorithm	Individual Risk Profile project
Finkelstein et al.^28^	2011				✓		HAT DSS
Veloso^29^	2014				✓	Agent-based simulation model	
Hillert and Stawiarz^30^	2015				✓		SMSreg
Reddel et al.^31^	2019				✓		AMS3 CDSS

Classification tasks have motivated a number of experts to implement DSSs particularly to be used in the MS domain. Esposito and De Pietro^18^ developed an ontology-based fuzzy DSS to assist neurologists in classifying MS lesions, i.e., white matter lesion (WML). They performed their study on a dataset that contained brain MRIs of 120 patients between 20 and 63 years old with clinically definite MS. The methodology of this DSS relied on a knowledge-based mechanism that integrated ontologies (to elucidate the structure of the knowledge semantically and to formulate clear outputs) and fuzzy logic (to comprehend the dataset's uncertainty and fuzziness) as knowledge representation techniques to embed an expert's high-level medical knowledge into the DSS. The DSS combined the obtained knowledge, in terms of fuzzy rules and ontologies, with Fuzzy Inference Ruled by Else-Action, i.e., the FIRE method. As a matter of fact, this methodology comprised three stages: knowledge elicitation, knowledge representation, and knowledge reasoning, respectively. Thereby, this DSS was able to classify WMLs and to obtain measures of their volumes. The authors argued that their proposed DSS provided better outcomes for patients with large lesions compared with patients with small lesions. They supported their argument by evaluating the performance of this DSS using the area under ROC curve (AUC) and Similarity Measures. The result of the AUC ranged between 0.82 and 0.87. Using different thresholds (0.25, 0.50, and 0.75), the similarity measures got the following results: 0.72–0.97 for the similarity index, 0.67–0.97 for the overlap fraction, and 0.01–0.37 for the extra fraction.

De Falco et al.^19^ proposed a DSS that utilized Differential Evolution (DE), an evolutionary algorithm, for automating the classification of potential MS lesions. This work used brain MRIs of 120 confirmed cases of MS. The methodology of this DSS extracted explicit knowledge, a set of explicit IF-THEN rules (i.e., classification rules), from the data in use. Furthermore, the methodology proceeded by finding separately the best set of rules for each class. The best set of rules at the end of the evolution emerged, so the classification here was all about searching for the optimal specification among others. As a result, this study reported a set of rules obtained in the 12th run for fold 3 as the best set of rules for the MS cases. The authors of this research used accuracy (81.21% over the training set, 85.92% over the testing set, and 81.68% over the whole dataset), sensitivity (82.06% over the training set, 88.18% over the testing set, and 82.64% over the whole dataset), specificity (78.69% over the training set, 80.25% over the testing set, and 78.87 over the whole dataset), and AUC (79.00% over the training set, 80.72% over the testing set, and 79.19% over the whole dataset) as an evaluation strategy to back their effort.

Siddiqui et al.^20^ established a design of an intelligent medical DSS for classifying brain MRIs as normal or abnormal. The primary motivation behind this design was to introduce a generalized DSS that can operate efficiently and effectively on various brain MRI datasets associated with neurological disorders. The researchers here ran their DSS against two datasets that consisted of T1- and T2-weighted brain MRIs of 340 right-handed patients diagnosed with major brain disorders, including brains affected by MS. In addition, the datasets also covered the patients’ demographics and clinical details. This DSS took the advantage of the discrete wavelet transform (DWT), principal-component analysis (PCA), and the least-squares support vector machine (LS-SVM) approaches to secure the goal of this study. The methodology of this work started with utilizing DWT in the feature extraction phase. Then, PCA performed feature reduction. The last step was to train the LS-SVM classifier by using the extracted reduced features. The authors claimed that their DSS classified the human brain as healthy or diseased with promising accuracy. Accordingly, their experiment yielded better results and outperformed other classifiers regarding sensitivity (100%), specificity (100%), and accuracy (100%). Moreover, the result proved that the DSS has a notable generalization ability.

Esposito et al.^21^ implemented an evolutionary-fuzzy DSS to support neurologists by recognizing MS lesions to evaluate the health status of individuals affected by MS. They conducted their experiment on the same dataset presented in Esposito and De Pietro^18^, ^19^ and De Falco et al.^18^, ^19^ Essentially, the methodology of this work composed knowledge representation, knowledge reasoning, and knowledge tuning, respectively. Knowledge representation interpreted and encoded the required medical knowledge of experienced clinicians in terms of linguistic variables, linguistic values, and IF-THEN rules. Knowledge reasoning specified a fuzzy inference technique that fitted the structure of the knowledge used for medical inferences. Knowledge tuning adopted DE to tune the knowledge through membership functions optimization for each linguistic variable involved in the rules ultimately to achieve the highest correct classification rate. This system obtained the best outcomes exceeding several classification algorithms compared with it in the study’s literature. To aid this finding, the authors of this study evaluated and compared their DSS’s accuracy, sensitivity, and specificity with several classification techniques, namely machine learning (ML) algorithms. The study recorded the average results over the 10 folds (accuracy on the training set was 89.10%, accuracy on the testing set was 88.79%, sensitivity was 0.88, and specificity was 0.88) and the results for the best fold in terms of the highest percentage of accuracy on the testing set (accuracy on the training set was 88.71%, accuracy on the testing set was 92.93%, sensitivity was 0.96, and specificity was 0.84). Moreover, the study reported the 10-fold classification accuracy of the proposed system (88.79) and compared it with other classifiers.

Easing the diagnostic procedure has encouraged several researchers to model DSSs. Linder et al.^22^ discussed proof of principle study by demonstrating the use of a computer-assisted decision (CAD) support that aimed to diagnose MS patients. The idea here revolved around the ability to distinguish between 73 MS patients, 22 patients with other chronic inflammatory diseases (CIDs) of the central nervous system, and 12 psychiatric patients (control group) in terms of cerebrospinal fluid and blood findings (i.e., standard laboratory findings). In other words, this CAD facilitated MS diagnosis by discovering any significant differences between MS patients and the other two groups of patients (MS versus CID and MS versus control group) based on the major parameters of the standard laboratory findings. To obtain the desired results, the authors here made the use of univariate and multivariate analyses using multiple logistic regression (MLR) and artificial neural networks (ANNs). MLR categorized patients based on their characteristics while ANNs performed feature selection on all parameters of the standard laboratory findings specified in the study. As a result, CAD was able to differentiate between MS patients and the control group. In comparison, CAD lacked the ability to deliver meaningful decision support when differentiating MS and CID patients since it did not disclose common parameters. Sensitivity, specificity, and accuracy assessed the performance of CAD as an eligible DSS. Noteworthy, the study evaluated the parameter sets MLR2 and MLR5 (MLRs with two and five parameters, respectively) and the ANN. The ANNs were able to perform with 84.9% sensitivity, 54.5% specificity, and 77.9% accuracy when differentiating MS and CID patients. Similarly, The MLR2 and the MLR5 recorded, respectively, 94.5% sensitivity, 22.7% specificity, and 77.9% accuracy (for MS versus CID). Furthermore, The ANNs distinguished MS and CID patients with 95.9% sensitivity, 66.7% specificity, and 91.8% accuracy. Likewise, The MLR2/MLR5 had 94.5%/95.9% sensitivity, 75.0%/83.8% specificity, and 91.8%/94.1% accuracy (for MS versus control group).

Pourakbari et al.^23^ designed a DSS suitable for diagnosing MS as early as possible. The study reported that the analysis of postural impairment, a type of quantitative movement disorders, was valid for detecting MS, even in its early stages, and for managing the disease before its severity progression. This study recorded the movement signals of 14 MS patients in the early stages (able to walk without an assistive tool) with an age range of 21–53 years. Also, the medical examination in this work documented the postural behaviors of 20 healthy subjects with an age range of 20–60 years to compare their results with MS patients. This DSS used image-processing algorithms to calculate the postural oscillation rates as spatial signals. By obtaining proper features (via statistical analysis) from these signals, this method separated control subjects from patients. However, the authors of this work did not evaluate the performance of their DSSs.

Dogan and Duru^24^ created a DSS for physicians by using image processing, and supervised and unsupervised ML algorithms, to detect lesions for diagnosing MS. Furthermore, the study compared the functionality of two types of ML tasks (supervised and unsupervised) concerning the objective. The presented techniques analyzed a dataset, collected by Loizou et al.,^32^ containing brain MRIs of 38 MS and clinically isolated syndrome (CIS) patients with average age equal to 34.1. For the methodology, the linear SVM was the supervised ML algorithm in use while k-means (with k = 4) acted as the unsupervised ML algorithm. The outcomes were acceptable and promising especially for SVM regarding the segmentation process. This is because k-means relied on objective assignment compared with SVM, which benefited from spatial coordinates of data. Calculating the result accuracy for both ML algorithms was the only evaluation mechanism presented in this work (70.24% for k-means and 91.04% for SVM).

Almasi et al.^25^ depicted a DSS prototype that aimed to minimize the time required to diagnose MS with the help of appropriate artificial intelligence (AI) techniques. In this work, the authors adopted two AI methods: case-based reasoning and rule-based reasoning. This work appeared to be limited as it lacked the experimental data and the design evaluation that support the researchers’ arguments.

Predicting the course of MS has attracted the attention of several researchers. For instance, Tintore et al.^33^ presented a notable paper that addressed MS prediction by analyzing the most common demographic, clinical, radiological, and biological features that have a strong correlation with the prognosis of MS. This study used a multivariate approach in the experiment and successfully stated that demographic characteristics, oligoclonal bands presence, and brain MRIs are considered as the impact prognostic factors, ordered from the lowest to highest impact factors, respectively. However, this study did not use DSS technology.

Several studies elaborate on the application of DSSs to predict optimally the probability of MS occurrence and progression. Veloso^26^ demonstrated the use of an agent-based simulation model that aimed to aid healthcare providers with a simulation tool. This tool was able to predict long-term disability and treatment effects on individuals affected by MS. For testing the methodology, a model that populated 100 virtual agents simulating patients with RRMS was created. Despite that, the validation task of this tool used real data from a group of 50 patients diagnosed with RRMS that lasted for at least 10 years. The dataset used was selected from a total of 173 patients. The author concluded by claiming that this simulation model can be used in everyday clinical practice for monitoring the disability episodes as it might scale for an individual with RRMS over 30 years. Aside from this, the tool was able to perform the treatment effect assessment over the same time frame. Because of the lack of real medical data for experimental use, evaluating the performance of the model was not presented.

The Sylvia Lawry Center for Multiple Sclerosis Research (SLCMSR)^27^ presented the "Individual Risk Profile" project that aimed to accurately predict short-, mid-, and long-range prognosis of MS during the lifetime of an individual affected by any type of this disorder. This project consists of an online analytical processing (OLAP) tool that uses a comprehensive database (contained data of 20,000 patients pooled from the academic and corporate sources), which is available to practitioners experienced in MS with an interest in clinical trials for decision-making purposes. To conduct this study, the researchers here derived only the data of 1,059 patients from the comprehensive database. The OLAP tool applied a matching algorithm as a strategy to match the patient of interest with all similar patients retrieved from the database. By doing so, the tool was able to predict the course of MS during the lifespan of the patient of interest by determining the disease course of all patients in the database that are similar to the patient of interest. The developers of this project argued that this work has potential advantages compared with purely model-based predictors. However, they also discussed that this tool needs improvements as it presented several limitations, so they did not use any evaluation metrics to assess the performance of this tool.

Numerous pieces of research have manifested the use of DSSs to aid MS patients with treatment decisions. Finkelstein et al.^28^ discussed the blueprint of the Home Automated Tele-management (HAT) DSS for MS patients. This system took the benefits of the current technologies to provide MS patients with the most convenient therapy and exercise plans during the rehabilitation phase. Furthermore, The HAT system adopted the model of chronic disease care proposed by Wagner et al.^34^ The designers of this DSS stated that HAT was a successful pilot DSS with promising outcomes for MS patients. The system would enhance the quality-of-life and the awareness of MS patients by minimizing the frequency of doctor visits, allowing patients to self-observe their health frequently, educating them on this condition, and guiding them through exercise routines needed during the rehabilitation procedures. To this end, this work observed a standalone system that was tested and evaluated based on end-user opinions. Therefore, the study lacked previous data acquisition, algorithm modeling, and performance evaluation.

Veloso^29^ proposed a web-based computer prognostic simulation model that addressed the needs to start/modify treatment plans, the transformation likelihood of a patient with CIS to definite MS, the long-term prognosis of MS, and the level of disability associated with MS progression. This simulation model applied, reformulated, and extended the simulation model presented by Veloso^26^ with the usage of distinct algorithms. This simulation model used two sets of data. First, the author obtained a dataset from reference studies dealing with the natural history of MS for experimenting on the proposed model. The researcher then used a dataset of 50 patients who had been living with RRMS for at least 10 years to validate the simulation process and its result. The study conductor proclaimed that this model answered the patients’ fundamental questions about their current state with MS at various evolutionary stages during the disease course. Finally, this work showed no evidence regarding the performance evaluation of the model.

Hillert and Stawiarz^30^ presented a review article that demonstrated The Swedish MS registry (SMSreg). SMSreg was developed as a web-based system to help all Departments of Neurology across Sweden. This system functioned as a decision support tool. Plus, it provided practitioners with patient information needed at clinical visits. SMSreg included data on 14,500 patients and recruited the data of 1,000 new MS patients reaching coverage of almost 80% throughout the country. As a decision support tool, SMSreg was valuable as it summarized the information needed to make decisions concerning disease-modifying drugs. In addition, this tool offered the ability to make decisions by comparing similar patients. Nevertheless, there was not much to say about the framework of the methodology of this system nor about whether it got evaluated.

Reddel et al.^31^ explained the idea of using alemtuzumab in MS Safety Systems (AMS3). AMS3 was developed as a CDSS to determine and organize MS patients’ important care routines, such as identifying risks associated with alemtuzumab therapy, scheduling periodic tests, sending reminders when needed, and analyzing test results, just to name a few. The study used a dataset that included a total of 10 patients with active RRMS receiving alemtuzumab treatment. The authors described the system’s overall architecture without describing in more detail the structure of the methodology, so this CDSS was evaluated based on its acceptance within the healthcare community. The designers of AMS3 argued that this CDSS accomplished the expected result.

Ultimately, based on the above-mentioned literature analysis, the use of DSSs in the MS field is quite limited due to the rarity of this medical condition. However, decision making is a critical task in MS practice. Researchers nowadays are applying several technologies, alone or together with DSSs, to support the process of decision making in the medical routine of MS. To keep up with this matter, the scope of this review was expanded to cover a specific number of technologies associated with decision making in MS studies. As noted, knowledge-based and ML approaches often form the basics of the DSS methodologies analyzed previously. Therefore, the next subsections outline the decision-making process in MS care using ontologies, the knowledge-based approach of interest, and ML algorithms.

### The Uses of Ontology in MS Research

The semantic web is an extension of the current World Wide Web that gives the information well-defined meaning, so the contents of the web become both machine-readable and human-readable.^35^ To make this possible, a key component of the semantic web, i.e., ontology, would offer a structured representation of the semantics that is relevant to one or several knowledge domains.^36^ An ontology is an explicit and formal specification of the shared conceptualization of a domain by means of classes, instances, properties, and semantic relationships.^37^ Capturing and personalizing knowledge (e.g., knowledge about chronic disorders) in formal, simple, powerful, and incremental ways and then applying appropriate reasoning processes to the personalized captured knowledge would be a remarkable result in biomedical domain research.^38^ Such an idea would be phenomenal in biomedicine as it reinforces sharing and reusing medical knowledge among health practitioners for decision-making purposes. This applies to MS as it is a chronic disorder. Existing publications about ontologies that serve the process of decision making related to MS are recapped next.

Hadzic et al.^39^ created a Mental Health Ontology (MHO) for deriving knowledge that aimed to prevent, diagnose, and treat and control mental disorders using data-mining algorithms to expose the patterns in mental health data. MHO consisted of subontologies representing mental disorder types, factors causing a specific type, and treatments suitable for a certain type. According to the findings of this work, MS was a physical factor affecting mental health because it may result in mood disorders.

Alfano et al.^40^ developed an ontology and a rule-based system that can automatically measure the load of the brain lesions (especially those that cannot be assessed visually) of MS patients. This was useful in terms of monitoring responses to treatments and studying the level of progression during the course of MS. This work utilized ontology as a knowledge representation model while the rule-based system acted as a reasoner to infer a new set of knowledge. To test the reasoning process, the proposed method used the MRI data of an MS patient. The authors stated that their approach showed greater sensibility as it recognized more lesions compared with an algorithmic procedure.

Jensen et al.^41^ originated Neurological Disease Ontology (ND), which aimed to formally structure common and accurate representation of a variety of neurological diseases with more specifics for MS and Alzheimer disease for clinical applicability and research purposes. The ultimate goal of ND was to represent each disease along with its genesis (genetically or environmentally), etiology, symptoms, syndromes, progression levels, diagnostic criteria, treatments, and relationships with other neurological disorders as a means to maximizing the potential reasoning capability. Up to the date of this work, this ontology contained nearly 450 classes in addition to over 700 classes imported from external ontologies.

Malhotra et al.^42^ proposed MS Ontology, which is specific for clinical and translational research related to MS. MS Ontology used a conceptual hierarchy to represent medical knowledge specific to MS, which was retrieved from scientific literature, database sources, and electronic medical records. Moreover, this ontology identified a huge amount of data that define the associations between risk factors, molecules, therapies, and several other diseases, aiming to improve societies’ understanding of MS. The authors argued that MS Ontology could acquire a wide range of MS concepts. They supported their argument by claiming that MS Ontology-obtained knowledge more accurately compared with PubMed advance searches.

All of these ontologies formulate knowledge bases that represent the domain knowledge of MS. Several reasoning and inference engines can make good use of expert knowledge captured in knowledge bases to automate the decision-making process, i.e., knowledge-based systems. This would enhance decisively medical practice for MS and possibly equalize or even outperform the medical justification of qualified MS specialists.

### The Utilization of Machine Learning in MS Studies

ML is an AI major discipline that draws attention toward its ability to learn patterns from input data using an increasing variety of algorithms (supervised and unsupervised) dedicated to automating the observation process that overcomes real-world challenges.^43^ Supervised ML algorithms train models using previously determined information (i.e., input data) representing class labels to automatically classify new objects or data not seen before.^44^ In contrast to supervised algorithms, unsupervised ML algorithms do not require previous information about the class labels as they train models to discover hidden structures and patterns (i.e., determine class labels) from unlabeled target variables.^45^ ML gains momentum in the medical realm for mining and analyzing large collections of medical data.^46^ In fact, healthcare organizations, as with most public and private organizations, have begun to apply ML as a central phase for analyzing medical knowledge for decision-making purposes.^47^ Notably, MS experts have adopted ML techniques mainly to distinguish MS from other pathologies and to investigate crucial characteristics of MS during its course.^48^ The vague patterns of MS (e.g., in terms of etiology, progression, clinical presentation, and response to drug therapies) elevate ML algorithms as the optimal set of tools that automate the recognition of patterns and regularities in MS data. An overview of articles that describe the use of the ML algorithms that yield better decision making regarding classification, diagnosis, and detection of MS are demonstrated next. To keep things simple, it should be noted that this overview is limited to articles of interest published during the last 5 years.

The success of ML has given the opportunity to pioneer algorithms able to provide a better classification of MS. Zurita et al.^48^ developed SVM classifiers able to recognize brain areas (affected by MS) that may assist to better diagnose potential cases of RRMS. This experiment used a dataset containing diffusion tensor imaging (DTI) and resting-state functional MRI (rsfMRI) data of 107 RRMS patients and 50 control subjects. Using 12 well-constructed rsfMRI- and DTI-based linear SVM learners, the authors here stated that these classifiers reliably discriminated (were able to avoid bias and overfitting) between RRMS patients and control subjects with accuracies up to 89%.

Lopez et al.^49^ utilized an unsupervised ML algorithm to cluster MS patients based on their genomic similarity and potentially discover valuable differences among these clusters. This algorithm clustered instances of a dataset that contained DNA samples from 191 MS patients. The methodology of this work used an agglomerative hierarchical clustering algorithm with multiple linkage methods to identify underlying cluster structures with the help of the majority vote approach. In addition, the methodology used a Silhouette index as an internal validity metric to select the appropriate number of clusters. The outcomes of this study revealed that the methodology presented here was able to identify patient clusters genetically without specifying the number of clusters in advance or indicating any previous input parameter. According to the authors, this methodology outperformed others found in the study’s literature regarding overfitting, as it had a significant Rand index greater than the benchmarked methods.

Ion-Mărgineanu et al.^50^ built multiple binary classifiers to automatically differentiate between patients with different clinical forms of MS. Namely, the researchers performed nine binary classification tasks for different combinations of MS types. This work used clinical data, lesion loads, and metabolic features of 87 MS patients, and 18 individuals served as healthy control subjects. The idea of this work was to compare the outcomes of the linear discriminant analysis (LDA), SVM with a radial basis function kernel (SVM-rbf), and random forest models. The results of this work showed that SVM-rbf, trained on clinical data and lesion loads, was the best classifier for distinguishing CIS from RRMS (F1 score = 71) or RRMS plus SPMS (F1 score = 72). Nevertheless, LDA, trained with clinical data, performed better when discriminating RRMS from PPMS (F1 score = 85) or SPMS (F1 score = 84).

Wang et al.^51^ aimed to segment MS lesions and non-specific white matter (NSWM) lesions separately based on their shape and spatial location features by adopting a spherical harmonics descriptor using an ML pipeline. To perform the experiment, the authors obtained two datasets. The first dataset contained 234 MS lesions and 190 NSWM lesions. The second dataset included 160 MS lesions and 119 NSWM lesions labeled by location. The authors trained three different ML models: logistic regression, SVM, and boosting tree (XGBoost). The authors continued by arguing that the proposed pipeline successfully classified MS and NSWM lesions with good accuracy (70.52%–87.97% for the logistic regression, 70.29%–74.89% for the SVM, and 85.58%–90.43% for the XGBoost) and AUC (83.76%–95.42% for the logistic regression, 70.49%–87.01% for the SVM, and 93.45%–96.43% for the XGBoost).

Automating the process of differentiating stable from potentially evolving MS cases is a research topic highly in demand. Salem et al.^52^ integrated intensity- and deformation-based approaches for automatically detecting new T2-w lesions. The study used a dataset that consisted of images from 60 different patients with CIS or early relapsing MS, with 36 of them being confirmed MS cases due to the appearance of new T2-w lesions in their scans. This work used a deformation-subtraction-based logistic regression model, i.e., a logistic regression algorithm that adopts the deformation field (DF) aspect, to detect new T2-w legions inside the white matter region. The authors declared that there was a significant difference in the model’s performance when including DF as it turned out that it improved the model’s accuracy. In fact, the combination of DF and logistic regression helped to boost the performance when detecting new T2-w lesions. To uphold this finding, the researchers compared their model with two state-of-the-art approaches and three variants of their model with fewer features. Their full model outperformed all the other models and had the best values for all the evaluation measures (sensitivity [74.30 ± 28.70], specificity [11.86 ± 18.40], and dice similarity coefficient [0.77 ± 0.23 for detection and 0.56 ± 0.23 for segmentation]) except when detecting very small lesions.

Zhang et al.^53^ reported a study that compared the performance of three ML algorithms with the intention to detect MS in the brain by using stationary wavelet entropy. The authors conducted their experiment on a dataset that included brain images of 38 MS patients and several healthy control subjects (the population of healthy controls is not specified). The authors applied three ML algorithms: decision tree (DT), k-nearest-neighbor (KNN), and SVM. The experiment recorded KNN as the best performer in terms of specificity (99.32%), precision (99.09%), and accuracy (97.94%), while the SVM performed the best in sensitivity only (97.34%). In contrast, the evaluation results of DT were the worst in all of the four measures. Thus, KNN yielded the best classification performance among the three algorithms in this detection process.

McGinnis et al.^54^ proposed a technique for estimating walking speed using a wearable device. The researchers of this proposal used accelerometers worn in several body locations to characterize gait speeds. To compare their mobility capabilities, this work recruited 30 subjects diagnosed with MS and 7 healthy controls. The authors utilized support vector regression (SVR) models to measure walking speed features indicated from the wearable accelerometer. In addition, the authors analyzed the correlation between speed estimation accuracy and device location combinations. They clarified that placing additional accelerometers in proximal locations would improve the accuracy of estimating the gait speed. The authors concluded their observation by claiming that there was a high correlation between the ground truth and estimated speeds during comfortable walking tests.

To better understand patterns that may underlie cardinal factors of MS, several recent studies strive to infuse ML into MS research. In light of this fact, ML algorithms are adopted excessively to automatize MS practice. The above-mentioned studies show how ML benefits MS research. It is important to mention that ML studies conducted for MS prediction (e.g., predicting MS progression), treatment, and diagnosis are beyond the scope of this review because they will form the baseline of future work.

## Discussion

In the preceding sections, MS studies were reviewed and analyzed about their usage of DSSs, ontology, and ML. Each of these disciplines, along with their underlying technologies, has certain benefits and drawbacks yielding the applicability of automating MS diagnosis, detection, treatment prescription, classification, and prediction. By virtue of its nature as a preference-sensitive condition, MS specialists and patients participate more intensively in MS decisions, particularly regarding diagnosis and treatment. Generally speaking, the MS diagnostic procedure constrains all MS specialists to obey the guidance of the criteria of McDonald et al.^55^ in addition to performing the Expanded Disability Status Scale (EDSS)^56^ and clinical examinations (e.g., MRI and LP) to confirm MS cases. MS specialists usually detect MS activities by comparing initial diagnosis reports with follow-up reports. Nevertheless, these procedures require intensive knowledge and experience given the inconclusiveness in this medical condition due to the lack of consensus diagnostic procedures. Furthermore, the variety of MS drug therapies offers a range of potential benefits, but they may also tolerate life-threatening risks. In like manner, predicting and classifying MS during and before the course of the disease are very challenging due to the ambiguity in terms of MS progression and occurrence pattern. To fulfill the need for automated systems that could help to overcome these gaps, several pieces of research, described in the previous section, manifest the use of modern technologies, e.g., DSSs, for this essence with remarkable findings and high-performance metrics.

Knowledge acquisition and representation of an expert is critical in developing reliable knowledge-based DSSs used for MS clinical routines. For instance, studies^18^^,^^21^ have demonstrated the use of fuzzy logic to handle the uncertainty in MS. This, in turn, would accurize solid knowledge representation to perform more rational knowledge reasoning that is able to make valid inferences. Significantly, the work presented by Esposito and DePietro^18^ integrated two knowledge representation approaches, namely ontology in addition to fuzzy logic. The importance of applying ontology here was to provide a shared understanding of the MS domain, i.e., semantic interoperability. This mixture led to knowledge elicitation, knowledge representation, and knowledge reasoning and inference with reduced uncertainty. Similarly, the methodology proposed by Esposito et al.^21^ combined fuzzy logic with DE to represent, reason, and tune knowledge. DE optimizes a complex problem by improving a candidate solution iteratively, so that it finds the best set of rules that guarantees the best set of knowledge. This combination can obtain the most optimal result because it leverages MS uncertainty using the best set of knowledge and rules. It is worth mentioning that De Falco et al.^19^ formulated a DSS using DE that obtained explicit knowledge through an optimal set of rules. To find this set of rules, each class has its own rules that are used to recognize the class’s instances. At the end of the DE evolution, the optimal set of rules that is used for classifying instances to their corresponding classes, emerged to form the best classification specification. Similarly, the study conducted by Esposito et al.^21^ used the same DE mechanism demonstrated by De Falco et al.^19^ in the knowledge-tuning phase. In fact, DE was applied to achieve the highest correct classification rate by tuning the knowledge via membership function optimization for each linguistic variable involved in the rules. Remarkably, these pieces of research performed well in classifying MS lesions with significant evaluation metrics results.

Two studies that used simulation models are reported in this review. The study presented by Veloso^26^ used an agent-based simulation model that showed the prediction of disability and treatment effects. This model virtually populated 100 agents that simulated patients with RRMS. The beauty of this work is its ability to abbreviate 30 years of monitoring and observing the quality-of-life of RRMS patients. By the same token, the work presented by Veloso^29^ extended the previous methodology with a web presence and the utilization of distinct algorithms. This model's functionality was extended as it considered other forms of MS (CIS and SPMS) suggested to start/modify treatment plans, and evaluated medical prognosis in the long-term. After all, using such simulation systems in MS clinical practice would allow clinicians to prompt several potentially rapid and appropriate medical interventions before any complications arise in the medical status of actual patients.

Certainly, DSSs need to be implemented in such a way as to highly simulate MS specialists. To optimistically reach this objective, the underlying structure of DSSs should be able to learn new patterns by observing subsets of data to produce reliable decisions without human intervention. This goal would be possible with the help of ML algorithms. The CAD tool presented by Linder et al.^22^ used ANN and logistic regression. This work showed promising results. The logistic regression model was able to discriminate MS patients based on their features, while the ANN selected features that are correlated mostly with MS. Likewise, the study by Dogan and Duru^24^ compared SVM with k-means in the lesion detection task. This comparison was in favor of the SVM, although both algorithms performed the segmentation process acceptably with promising results. However, both studies need improvements in terms of applying the most suitable ML model by comparing the results of different ML algorithms. In a word, it should be noted that each ML algorithm performs differently depending on the problem and the dataset in use, so comparing the performance of several ML algorithms should be sufficient when adopting ML as the ground solution.

Commonly, the SVM algorithms have been used extensively in several studies presented in this review whether as a DSS basis or as standalone models. Some studies^20^^,^^24^^,^^48^^,^^50^^,^^51^^,^^53^^,^^54^ have been reported to rely entirely or partially on SVMs despite the fact that they are used for mutual or different objectives. For instance, the linear SVM was applied in the DSS of Dogan and Duru^24^ and in the experiment of Zurita et al.^48^ for diagnosing MS. On the contrary, the study established in Ion-Mărgineanu et al.^50^ used the SVM with an rbf kernel to segregate patients with different MS clinical forms. Furthermore, the standard SVM model was used as a part of an ML structured pipeline by Wang et al.^51^ and as an individual model of Zhang et al.^53^ for detecting MS. Moreover, another study by Siddiqui et al.^20^ utilized the LS-SVM classifier to classify brain MRIs as normal or abnormal. In addition, McGinnis et al.^54^ used the SVR model to measure walking speed features in a wearable device, which is used to characterize gait speeds to compare MS patient mobility. The comparison between all of the SVMs presented in this review seems imbalanced for two reasons. First, they are used for different objectives and with different datasets. Second, they are used partially as one of the essential tools of a DSS paradigm or entirely as an independent method. However, the performances of these SVMs can be explored despite the above contradictions. To emphasize this, the LS-SVM was the best SVM because it guarantees higher evaluation rates with minimum computation time and complexity even when it is running against huge datasets. The LS-SVM is an enhanced, reformulated, and upgraded version of the classical SVM. LS-SVM ensures more accuracy by using least-squares to modify and correct the classifier's behavior to minimize the errors, i.e., cost function. As stated in Siddiqui et al.,^20^ the performance of the model used in the study with the LS-SVM classifier that used the rbf kernel exceeded all other models, especially those that applied standard SVM with different kernel values. Yet, exploring and comparing the performance of several sets of parameters within LS-SVM is something that needs to be considered in future works that adopt LS-SVM to have the most optimal model performance.

It could be inferred from this review that the number of studies conducted to address the usage of DSSs in the MS field is quite limited. Indeed, the acceptance of DSSs within the MS domain remains limited. This condition is not getting proper attention compared with other incurable diseases, such as Alzheimer disease due to its uncommonness and data scarcity. Nonetheless, the direction of future work should incline toward applying DSS technologies, and potentially knowledge graph techniques, that are able to understand MS progression and occurrence patterns. Additional work should also adopt these machine-based approaches to emphasize MS etiology and long-term effects of MS on the quality-of-life of the affected individuals. Moreover, the correlation between MS and other disorders (especially chronic neurological and autoimmune diseases) should be investigated. In spite of this, MS’s intended researchers require an extensive amount of data, but access to them is very restricted. To overcome this issue, the FAIR principle for MS data^57^ should be considered in the near future. Considering these recommendations would enhance the clinical practice experience in MS.

## Conclusion

MS is a chronic neurological disease that affects the brain and the spinal cord. The decision-making process regarding this phenomenon is critical, and it is considered as shared decision making. Automatic solutions proposing optimal alternatives that could make the shared decision making easier are highly in demand in the field of MS. DSSs in the MS realm are very favorable, especially because of their ability to enhance the shared decision-making process. Recent studies demonstrated the increasing need for DSSs in the MS domain to aid decision makers to nominate the right decision among several alternatives. To our knowledge, the adaptation of DSSs in the field of MS is still insufficient.

The objective of this paper is to provide a general overview of the development of DSSs using different methodologies for improving the clinical practice experience in MS, with a special focus on the application of model-driven approaches. This overview is beneficial for those who need to better understand the common methodologies that the current DSSs use to support decision making in MS. All methodologies applied to develop the current MS DSSs have high efficiency in classifying, diagnosing, predicting, or treating MS. However, the efficiency of these methodologies is different with regard to their underlying technologies. In addition to DSSs, current research topics are applying several technologies to support the decision-making process in the MS domain. A knowledge-based approach and ML mechanisms are considered, among others, in this overview.

Future work should focus on applying DSS technologies to understand the progression and occurrence patterns of MS, to emphasize MS etiology, to highlight the long-terms effects of MS on the quality-of-life of the affected individuals, and to find the correlation between MS and other disorders. Considering these recommendations would enrich the field of MS. In conclusion, DSS technologies have the potential to be pragmatic in the MS domain and in MS research.
